# Contrasted Fitness Costs of Docking and Antibacterial Constructs in the EE and EVida3 Strains Validates Two-Phase *Anopheles gambiae* Genetic Transformation System

**DOI:** 10.1371/journal.pone.0067364

**Published:** 2013-06-26

**Authors:** Doug Paton, Anne Underhill, Janet Meredith, Paul Eggleston, Frederic Tripet

**Affiliations:** Centre for Applied Entomology and Parasitology, School of Life Sciences, Keele University, Keele, Staffordshire, United Kingdom; Johns Hopkins University, Bloomberg School of Public Health, United States of America

## Abstract

The deployment of transgenic mosquitoes carrying genes for refractoriness to malaria has long been seen as a futuristic scenario riddled with technical difficulties. The integration of anti-malarial effector genes and a gene-drive system into the mosquito genome without affecting mosquito fitness is recognized as critical to the success of this malaria control strategy. Here we conducted detailed fitness studies of two *Anopheles gambiae s.s*. transgenic lines recently developed using a two-phase targeted genetic transformation system. In replicated cage-invasion experiments, males and females of the EE Phase-1 docking strain and EVida3 Phase-2 strain loaded with an antimicrobial peptide (AMP) expressed upon blood-feeding, were mixed with individuals of a recently-colonized strain of the Mopti chromosomal form. The experimental design enabled us to detect initial strain reproductive success differences, assortative mating and hybrid vigor that may characterize mosquito release situations. In addition, the potential fitness costs of the unloaded Phase-1 and loaded Phase-2 genetic constructs, independent of the strains’ original genetic backgrounds, were estimated between the 1^st^ instar larvae, pupae and adult stages over 10 generations. The Phase-1 unloaded docking cassette was found to have significantly lower allelic fitness relative to the wild type allele during larval development. However, overall genotypic fitness was comparable to the wild type allele across all stages leading to stable equilibrium in all replicates. In contrast, the Phase-2 construct expressing EVida3 disappeared from all replicates within 10 generations due to lower fitness of hemi- and homozygous larvae, suggesting costly background AMP expression and/or of the DsRed2 marker. This is the first study to effectively partition independent fitness stage-specific determinants in unloaded and loaded transgenic strains of a Phase-1–2 transformation system. Critically, the high fitness of the Phase-1 docking strain makes it the ideal model system for measuring the genetic load of novel candidate anti-malarial molecules *in vivo*.

## Introduction

There has been a growing focus on the practical implementation of releasing transgenic mosquitoes as a means of disease control as the technological and methodological hurdles of achieving efficient transgenesis and developing gene-drive systems capable of spreading effector genes into target populations look to be overcome in a very near future. The recent release of transgenic sterility-inducing mosquitoes in both semi-field conditions in Malaysia [1] and full field trials on Grand Cayman [2] is fuelling expectations that mosquitoes refractory to dengue and malaria could soon be deployed. Recent milestones such as increasingly efficient transformation protocols [3], newly characterized expression systems [4], coupled with the announcements of both a functional homing endonuclease-based gene drive system [5] and a rapidly expanding repertoire of potential anti-malarial effector genes [6] suggest that we are better placed than ever to develop a system for driving transgenic disease refractoriness into wild mosquito populations.

All transgenic control strategies rely on genetically-modified male mosquitoes being able to successfully compete with wild males for mates once released in the field. In the case of population replacement strategies, the male and female F_1_ progeny and subsequent generations carrying transgenic constructs post-release must also be vigorous, fecund and robust enough to ensure the continuing spread of these genes through the target population. Thus, assessing the fitness and mating competitiveness of transgenic lines, but most critically of the transgenic alleles once they spread within the wild type population is a vital step in the development of functional transgenic mosquitoes for the control of malaria transmission.

There are a number of ways in which transformation could potentially affect fitness (reviewed in [7]). Firstly the strong expression of exogenous genes may reduce the competitiveness of a transgenic individual by having a deleterious behavioral or physiological effect as it accumulates in tissues (e.g. [8]), or simply by imposing an additional metabolic cost on the transgenic not suffered by a wild type competitor (e.g. [9]). Secondly, and independent of transgene expression, the site at which a transgenic construct integrates into the target genome can itself have a significant effect on fitness. For example, the transgene may integrate into the open reading frame or regulatory sequence of an endogenous gene, thus interrupting its function and leading to fitness costs or even recessive lethality (e.g. [10]). Thirdly, the process by which a transgenic lineage is created necessarily involves at least one - and in some cases two - severe genetic bottlenecks where a single mosquito is the progenitor of the entire subsequent population of transgenic insects, leading to inbreeding depression and fixation of deleterious recessive alleles by random genetic drift. This effect can be, theoretically, ameliorated by successive generations of outcrossing to more genetically diverse populations. Finally, and depending on the site of integration and the genetic background of the mosquito, deleterious recessive alleles at loci proximal to the site of the transgene integration can - in a process known as hitchhiking - be positively selected for through tight-linkage with the transgene insert and may impose a fitness cost in homozygous individuals [7].

Evaluating the fitness of transgenic mosquito lines can be done in several ways. Direct comparisons of genetically-modified strains to their unmodified parental strain or a wild-type colony have been made in order to compare fitness components such as adult fecundity as well as developmental rates and survival at difference life stages. In theory, such comparisons do not allow partitioning of the fitness costs linked to the transgenic mosquito genetic background (e.g. inbreeding depression) from those linked to the genomic location of the transgenic construct or the expression of its effector molecules. However, since the properties of effector molecules - e.g. antiparasitic - are often tested on homozygous transgenic lines, direct mosquito fitness comparisons may serve to objectively identify grossly unfit homozygous lines that may not be worth further characterization. Direct comparisons have revealed strong fitness costs in terms of fertility and survivorship in transgenic lines of *Aedes aegypti* carrying an enhanced GFP gene or expressing transposase from the *Hermes* and Mos1 elements [10]. They also showed reduced size, survival and longevity in the OX513A line of *Aedes aegypti* that carries a tetracycline repressible, dominant lethal positive feedback system (RIDL) for the release of sterility inducing individuals [11]. The confounding effects of genetic background inherent to the direct comparisons approach are typically decreased by repeatedly backcrossing transgenic lines into a wild-type line in order to increase their heterozygosity prior to the experiments. For example, comparisons of non-transgenic and transgenic lines have revealed differences in fertility and survival between *An. stephensi* transgenic lines expressing active phospholipase A2 (PLA2), a component of bee venom, and non-transgenic lines suggesting a negative effect on their midgut nutrient absorption [8]. Further comparisons in *Aedes fluviatilis* expressing inactive PLA2 revealed no apparent negative effects of the protein, no difference in fertility, and even increased survival in some transgenic lines compared to non-transgenic ones [12].

A second approach for evaluating the fitness of transgenic lines that resolves some of the limitations of direct strain comparisons has been to compare the fitness parameters of individuals hemizygous for the transgene, with those of sibling wild-type individuals [13], [14]. Hemizygosity is achieved by first crossing homozygous transgenic with wild-type individuals and eliminates the confounding factors of inbreeding depression and potential costs of recessive alleles hitchhiking with the construct. Although this constitutes a vast improvement over direct homozygous strain comparisons, fitness costs that usually affect individuals homozygous for the transgene construct (i.e. recessive and co-dominant effects) cannot be measured. The lack of evaluation of transgene fitness costs in the homozygous state is made particularly obvious in studies that test the effects of antiparasitic effector molecules using homozygous individuals but transgene fitness costs on hemizygous ones [13], [14].

Finally, the fitness of the transgenic construct independent of the transgenic line's genetic background can be followed using cage-invasion experiments in which the transgenic allele is introduced into a wild-type population and its frequency monitored over time [8], [9], [15], [16]. These experiments best simulate real release-like situations but require carefully planned and comparatively complicated design. The main advantages of such approaches are that: (1) they allow direct competition between transgenic and wild-type alleles; (2) they enable an assessment of the fitness of individuals hemi- and homozygous for the transgene (i.e. recessive, co-dominant, dominant effects); (3) several generations-worth of recombination breaks down the linkage between the construct and all but the closest recessive deleterious genes that may be hitchhiking with it. Depending on the design of the experiment, one can also assess the initial reproductive success of homozygous transgenic and wild-type individuals, potential problems associated with assortative mating amongst released homozygous transgenic individuals, and the importance of hybrid vigour in first generation hemizygous individuals. All of these aspects contribute to making cage-invasion experiments the most rigorous for assessing the fitness of transgenic strains but also the most useful in terms of generating the fitness parameters required for population dynamic models of the spread of transgenic alleles in target populations.

Only a handful of studies explicitly investigating transgenic mosquito fitness have described a fitness-neutral transformation that is stable in mixed populations over multiple generations. Cage-invasion experiments complementing direct strain comparisons allowed the identification of an *An. stephensi* line expressing SM1 whose transgenic construct subsisted in test populations for 5 generations [8]. Using the same approach, *Aedes fluviatilis* lines expressing inactive been venom enzyme PLA_2_ were shown to bear no apparent fitness costs [12]. However, most other studies investigating the persistence of a given transgenic construct over multiple generations have observed a rapid decrease in transgene frequency, and in some cases total extinction of the transgenic allele [8], [9], [10], [16].

Recent progress in the development of site-specific transgene integration systems in *Ae. aegypti* [17] and *An. gambiae* [3] can potentially provide the scientific community with the means to thoroughly evaluate the potential fitness of a whole suite of effector transgenes. Site-specific transgene integration relies on two steps of genetic transformation: Phase-1 uses transposon-mediated integration to create a so-called docking strain carrying a phenotypic marker and a site-specific *phiC31* integrase recognition sequence [18], whilst Phase-2 uses an endo- or exogenous integrase to introduce a second phenotypic marker and an effector gene at the docking site. The power of this approach lies in the possibility to efficiently produce and compare different loaded transgenic lines produced from a single well-characterized docking site. Having different effector genes and their promoter sequences located precisely in same location in the mosquito genome, effectively controls for variation in potential fitness costs caused by gene-hitchhiking, positional expression effects and the site of integration.

As a proof of principle, we set out to assess and compare the fitness of the unloaded Phase-1 EE docking strain and loaded Phase-2 EVida3 transgenic strain recently developed using the two-phase targeted genetic transformation system in *An. gambiae* s.s. [3]. Preliminary studies of the EE docking strain and the EVida3 strain, which expresses a tetramer of the synthetic AMP, Vida3 [19] under the control of the *An. gambiae carboxypeptidase* promoter, suggested that the two strains bred and survived well under standard laboratory conditions [3]. Here we performed replicated cage-invasion experiments to assess the long-term stability of the Phase-1 and 2 genetic constructs independent of their genetic background when competing against wild-type alleles. The design of the experiment allowed us to detect initial differences in reproductive success and assortative mating in the transgenic strains, as well as to evaluate the importance of heterosis in their F_1_ progeny. In addition, the potential fitness costs of the unloaded Phase-1 and loaded Phase-2 genetic constructs were estimated at the 1^st^ instar larvae, pupae and adult stages over 10 generations. The results highlight the power of cage-experiments for partitioning the different fitness costs potentially affecting genetically-modified alleles in a mosquito release context. The high fitness of the EE docking line provides researchers with the ideal system to test the potential genetic load of candidate transgenic constructs carrying effector genes targeting the malaria parasite or other mosquito traits affecting malaria transmission.

## Results

### Mating and Reproductive Success in the Initial Generation (F_0_–F_1_)

#### Assortative mating amongst strains

**Figure 1:**
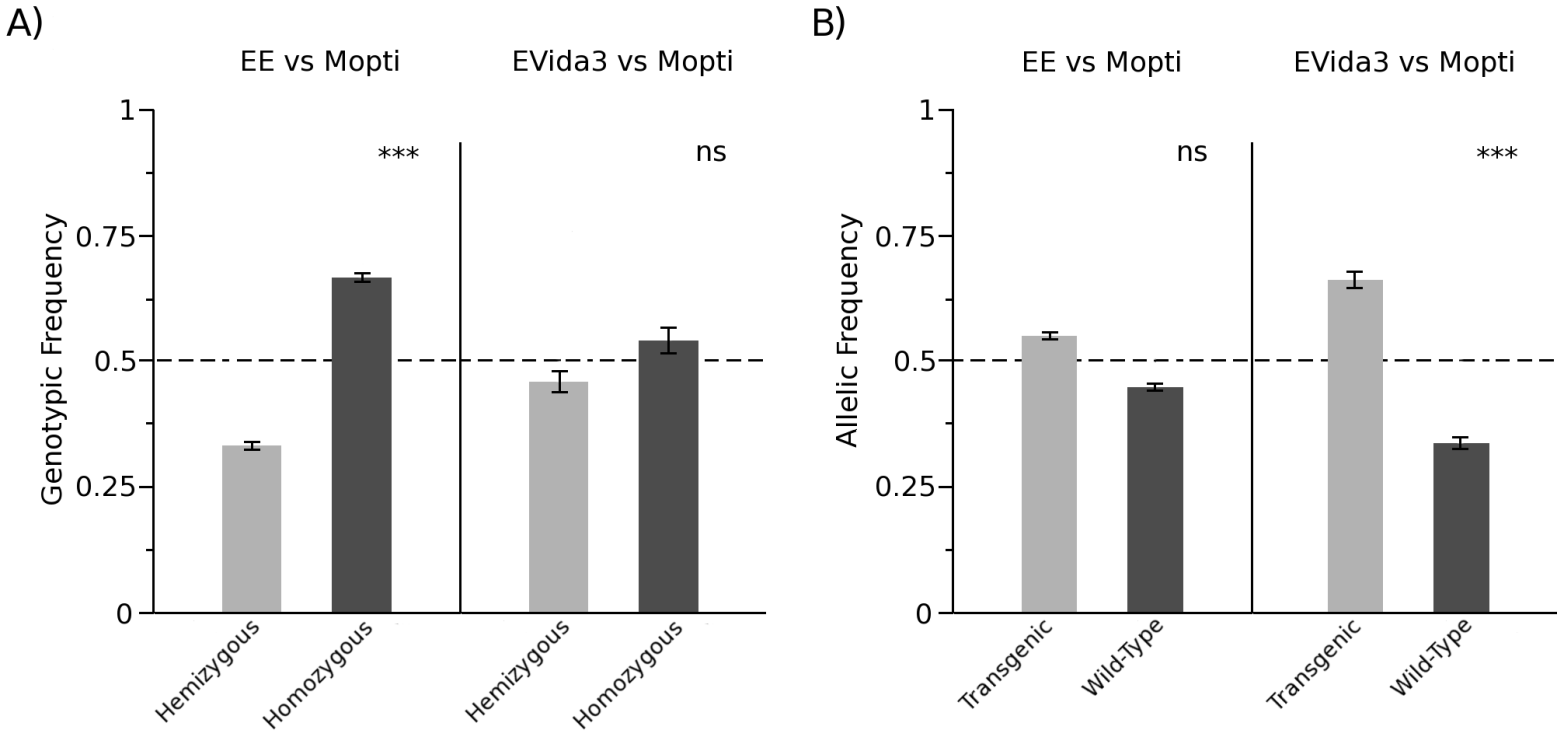
Assortative mating and reproductive success of the transgenic and wild-type lines. In A) the frequency of the hemizygote (TW) and homozygous (WW, TT) genotypes were compared in both the Mopti vs EE and Mopti vs EVida3 comparisons. In B) the frequency of the transgenic (T) and wild-type (W) alleles in both the Mopti vs EE and Mopti vs EVida3 comparisons were compared. Error bars represent 95% confidence intervals. Significance levels of a Chi-square test are indicated - ns, not significant, *: *P*<0.05, ** *P*<0.01, *** *P*<0.001.

Evidence of assortative mating in both experiments was tested by comparing the observed frequency of hybrids and homozygous genotypes in the L_1_ larvae (1^st^ instar) sample of the F_1_ progeny to those predicted given the equal numbers of homozygous males and females used at the start of each experiment (50∶50 ratio) (Table S1–2; Fig. 1A). Significant assortative mating was observed in the Mopti vs EE comparison over all replicates (Chi-square Goodness of Fit: *n* = 144, df = 1, *χ* ^2^ = 16.3, *P*<0.001) and within each replicate (*P*<0.05 in all cases). In contrast, significant assortative mating in Mopti vs EVida3 comparisons was only detected in replicate 2 (Chi-square: *n* = 48, df = 1, *χ* ^2^ = 5.4, *P* = 0.018) but was not significant over all replicates (*P*<0.05).

#### Reproductive success differences between strains (F0–F1)

The overall mating success of transgenic and non-transgenic lines, including the combined effects of male and female mating success and female fertility, was assessed prior to any recombination events by comparing the frequencies of transgenic and wild-type alleles in the F_1_ progeny (L_1_ larvae in both experiments) (Fig. 1B). In comparisons of Mopti vs EE no overall significant difference was found between the fitness of the two strains (Chi-square Goodness of Fit: *n* = 288, df = 1, *χ* ^2^ = 2.7, *P* = 0.099) nor within any of the replicates (*P*>0.152 in all cases). In contrast, in Mopti vs EVida3 comparisons, the EVida3 strain had higher initial fitness than the Mopti strain in the first and second replicates, leading to an overall significant difference across replicates (Chi-square: *n* = 288, df = 1, *χ* ^2^ = 29.9, *P*<0.001).

#### Hybrid vigor (F1)

**Figure 2:**
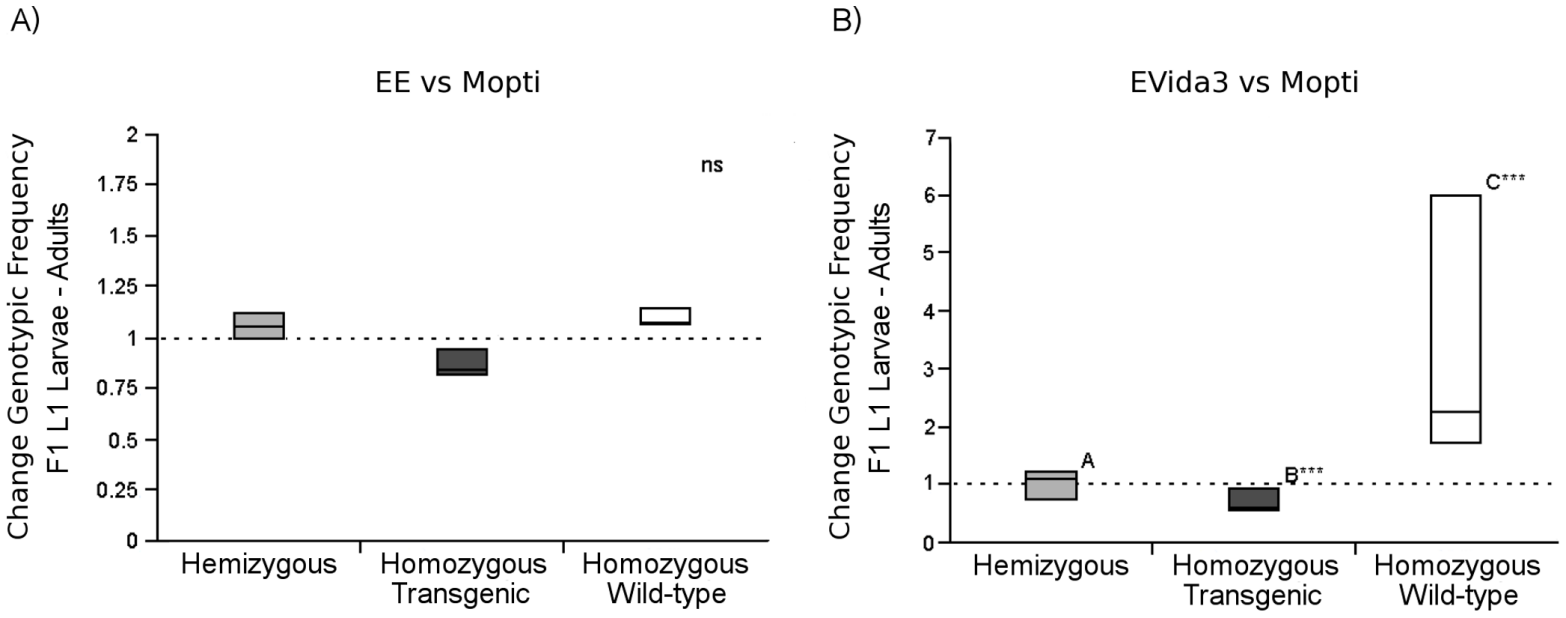
Test for hybrid vigor amongst the F1 progeny in EE and EVida 3 vs Mopti cage invasion experiments. In A) Change in frequency in hemizygotes, homozygous transgenic and homozygous wild-type individuals from F_1_ larvae to adults in comparisons of the wild-type Mopti allele to the transgenic Phase-1 EE allele; in B) to the Phase-2 EVida3 allele. Boxplots were median, quartiles and min-maximum values. Significance levels of a Chi-square test are indicated - ns, not significant, *: *P*<0.05, ** *P*<0.01, *** *P*<0.001.

Evidence of heterosis or hybrid vigor in the form of increased survival from larval to pupal and from pupal to adult stages was specifically tested by comparing the change of genotypic frequencies of hemizygotes and homozygotes between the F_1_ L_1_ larvae and F_1_ adult stages. In the Mopti vs EE comparisons (Table S1; Fig. 2A), there was no overall significant difference in changes in genotypic frequencies between hemizygous (TW) and homozygous transgenic individuals (TT) and homozygous wild-type (WW) individuals from larval to adult stages over the 3 replicates (Logistic regression: n = 288, replicate: df = 4, *χ^2^* = 2.47, *P* = 0.650, stage: df = 2, *χ^2^* = 0.756, *P* = 0.686).

In Mopti vs EVida3 comparisons (Table S2; Fig. 2B), the change in frequency of hemizygotes and homozygotes between the F_1_ L_1_ larvae and F_1_ adult stages significantly differed between genotypes and replicates (Logistic regression: n = 288, replicate: df = 4, *χ^2^* = 21.01, *P*<0.001, stage: df = 2, *χ^2^* = 14.0, *P*<0.001). Post-hoc comparisons show this was due to a significantly higher survival of homozygous WW individuals compared to homozygous TT (Marascuilo pairwise comparison: *χ^2^* = 7.46, *P* = 0.024) and hemizygous TW individuals (Marascuilo: *χ^2^* = 15.1, *P*<0.001) whilst the other two groups did not differ significantly (*χ^2^* = 2.27, *P* = 0.321). Thus, there was no evidence of a significant heterozygote advantage.

### Allelic and Genotypic Fitness in Further Generations (F_2_–F_10_)

#### Transgenic vs wild-type fitness comparisons

**Figure 3:**
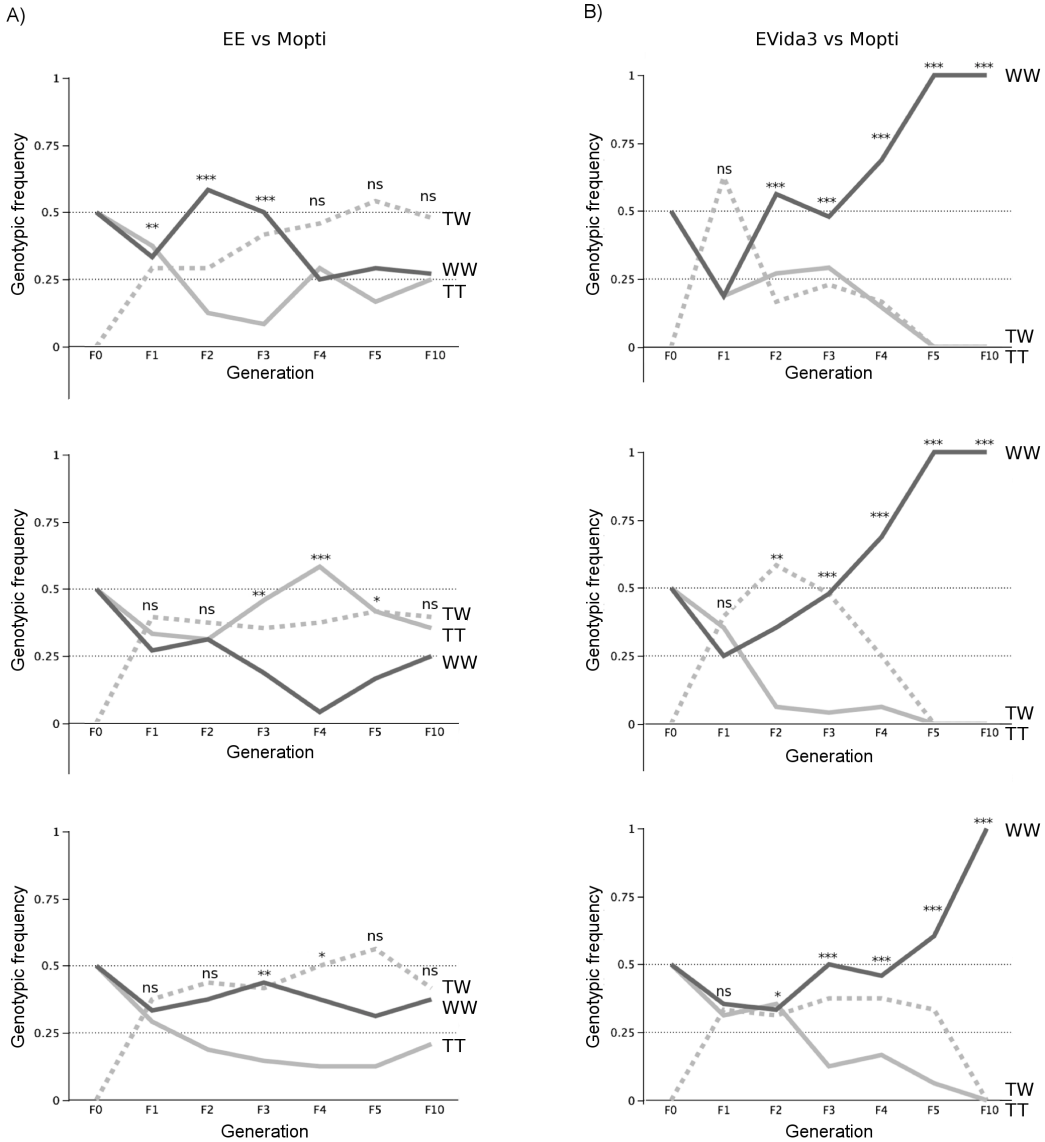
Frequency of hemizygotes, homozygous transgenic and homozygous wild-type genotypes over 10 generations in three independent replicates. In A) the frequency of individuals homozygous (TT) and hemizygous (TW) for the Phase-1 EE docking construct are compared to homozygous wild-type (WW); in B) the frequency of homozygotes (TT) and heterozygotes (TW) for the Phase-2 EVida3 construct are compared to homozygous wild-type (WW). Significance levels of a Chi-square test of HWE are indicated - ns, not significant, *: *P*<0.05, ** *P*<0.01, *** *P*<0.001.

Following mixing and recombination between the transgenic lines and the wild-type strain (Mopti) over 10 generations, the two transgenic elements exhibited strikingly different trajectories over time (Table S1–2; Fig. 3A–B). After 10 generations, the Phase-1 (EE) transgene was present in all 3 replicates of the Mopti vs EE comparisons (Fig. 3A). Despite some fluctuations between F_2_ and F_5_, by generation F_10_ the observed genotypic frequencies of TW, TT and WW did not deviate significantly from Hardy-Weinberg equilibrium (HWE) nor from the 50∶25:25 ratio predicted from starting conditions (Chi-square Goodness of Fit, *P*>0.05 in all cases). In contrast, the frequency of the Phase-2 EVida3 transgenic construct (Fig. 3B) decreased rapidly and was no longer detectable after 5 generations in two replicates, and by generation 10 in the third. Deviations from HWE frequencies and from a 50∶25:25 ratio were highly significant from the F_2_ onwards in all replicates.

#### EE vs EVida fitness comparisons

The frequencies of EE and EVida3 transgenic alleles competing against the wild-type Mopti allele were formally compared using logistic regression on the combined allelic frequency data of the 3 replicates from each type of comparison. As expected, transgenic allele frequencies were significantly higher in Mopti vs EE comparisons than in Mopti vs EVida3 (Logistic regression LR: *n* = 2880, df = 1, *χ* ^2^ = 77.6, *P*<0.001) and varied significantly between generations (logistic regression: *n* = 2880, df = 4, *χ* ^2^ = 65.5, *P*<0.001). Breaking down the analysis by generation showed that there was no significant difference in transgenic allele frequencies between the two experiments in generations F_1_ (Logistic regression: *n* = 576, df = 1, *χ* ^2^ = 0.0, *P* = 1.000) and F_2_ (*χ* ^2^ = 0.12, *P* = 0.734). However, from generation F_3_ (*χ* ^2^ = 5.4, *P* = 0.020), the frequency of the EE docking construct was significantly higher than that of the EVida3 cassette (*P*<0.001 in both F_4_ and F_5_ generations).

#### Life stage-specific fitness costs (F2–F5)

**Figure 4:**
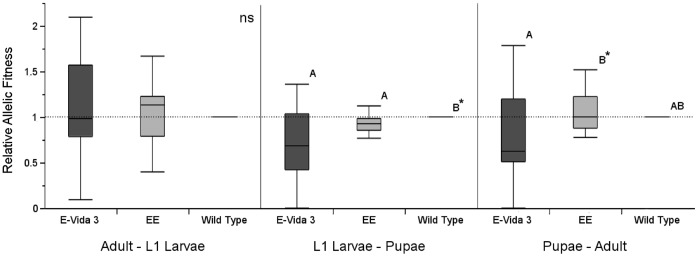
Allelic fitness for transgene alleles relative to wild-type for three independent developmental periods. Boxplots were median, quartiles and min-maximum fitness from adult to 1^st^ instar larvae of the next generation (Adult - L_1_ Larvae), larvae to pupae (L_1_ Larvae - Pupae) and pupae to adult (Pupae - Adult). Significance levels of pairwise Wilcoxon tests are indicated - ns, not significant, *: *P*<0.05, ** *P*<0.01, *** *P*<0.001.

Analyses of stage-specific fitness components in generations F_2_–F_5_ for the 3 replicates combined showed no significant reduction in fitness of the EE and EVida3 alleles relative to the wild type allele from adults to the next generation’s L_1_ larvae (Wilcoxon signed-rank tests: EE-Mopti: *n* = 12, *Z* = 10.0, *P* = 0.470; EVida3-Mopti: *n* = 12, *Z* = 4.0, *P* = 0.791). Similarly, there was no significant reduction in comparison to the wild type and during development from pupae to adults (Wilcoxon signed-rank tests: EE-Mopti: *n* = 12, *Z* = 7.0, *P* = 0.622; EVida3-Mopti: *n* = 12, *Z* =  -19, *P* = 0.148) despite EE having significantly higher fitness than EVida3 (Mann-Whitney: *n* = 24, Z =  −1.99, *P* = 0.046) (Fig. 4). However, allelic fitness relative to the wild type was significantly reduced in both the Phase-1 EE and Phase-2 EVida3 strains during larval development (Wilcoxon signed-rank tests: EE-Mopti: *n* = 12, *Z* =  −27.0, *P* = 0.034; EVida3-Mopti: *n* = 12, *Z* =  −23, *P* = 0.042) (Fig. 4).

**Figure 5:**
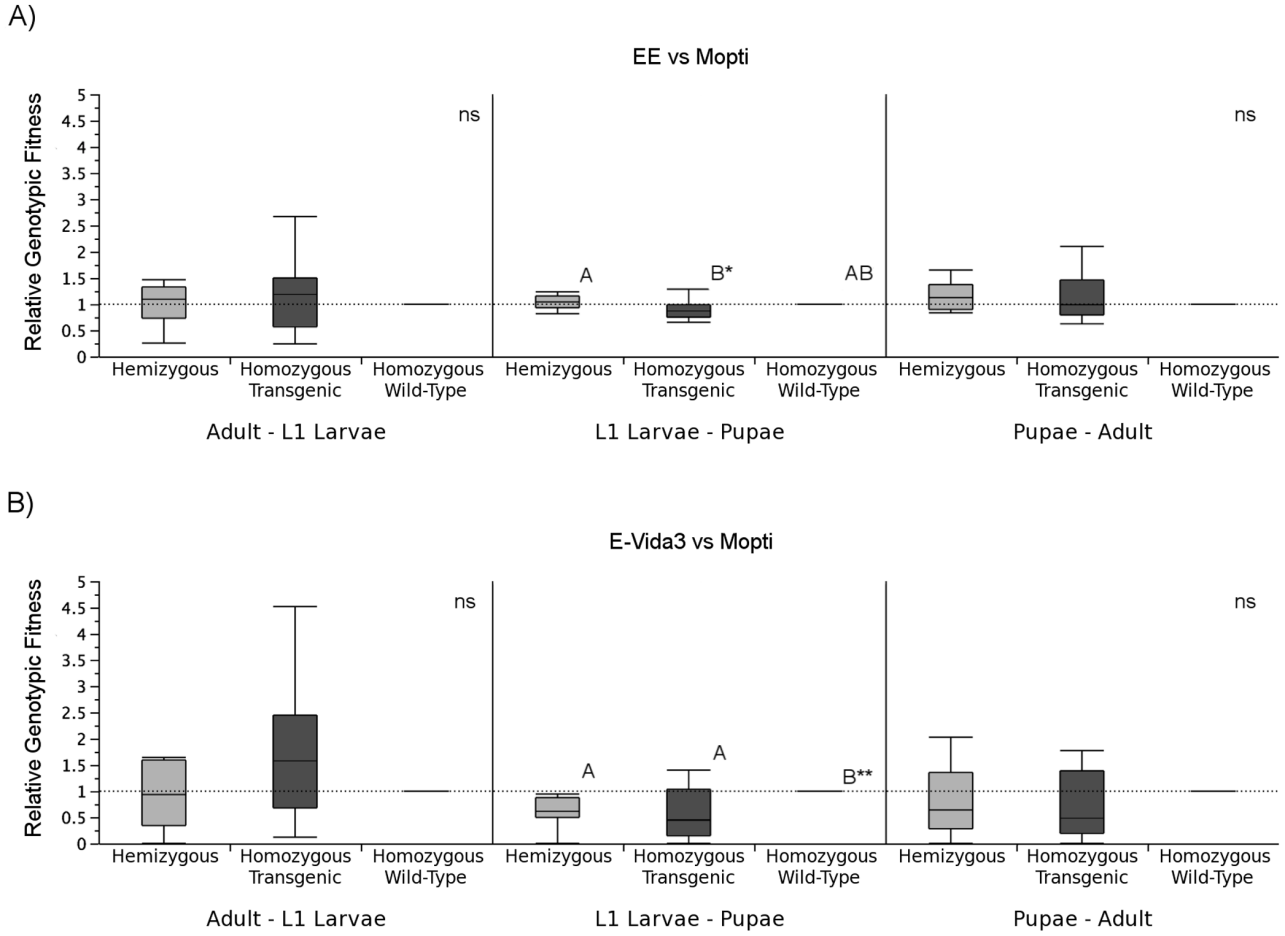
Genotypic fitness for hemizygotes and transgenic homozygotes relative to homozygous wild-type individuals over three developmental periods. In A) the fitness of the homozygous and hemizygous Phase-1 EE genotypes were compared to wild-type homozygotes; in B) the fitness of the homozygous and hemizygous Phase-2 EVida3 genotypes were compared to wild-type homozygotes. Boxplots were median, quartiles and min-maximum values. The significance levels of a pairwise Wilcoxon test are indicated - ns, not significant, *: *P*<0.05, ** *P*<0.01, *** *P*<0.001.

In Mopti vs EE comparisons no significant differences in genotypic fitness relative to the homozygous wild type were found in hemi- or homozygous transgenic genotypes from the adult to L_1_ larval stages (Wilcoxon signed-rank tests: TW-WW: *n* = 12, *Z* = 8.0, *P* = 0.569; TT-WW: *n* = 12, *Z* = 9.0, *P* = 0.519), L_1_ larvae to pupae (TW-WW: *Z* = 11.0, *P* = 0.353; TT-WW: *Z* =  −17.0, *P* = 0.148) or pupae to adults (TW-WW: *Z* = 20, *P* = 0.129; TT-WW: *Z* = 6.0, *P* = 0.664) (Fig. 5A). Hemizygous EE transgenics had significantly higher relative fitness than homozygous ones from L_1_ larvae to the pupal stage (Mann-Whitney: n = 24, Z =  −2.02, *P* = 0.043).

There was no significant difference in the larval developmental rate of the three genotypes as evidenced by the lack of changes in genotypic frequencies observed between the two pupal samples taken at a 3-day interval (logistic regression: *n* = 720, replicate: df = 4, *χ* ^2^ = 38.1, *P*<0.001; sample: df = 2, *χ* ^2^ = 0.27, *P* = 0.873).

In Mopti vs EVida3 comparisons there were again no significant differences in genotypic fitness relative to the wild type from adult to L_1_ larvae (Wilcoxon signed-rank tests: TW-WW: *n* = 12, *Z* =  −3.0, *P* = 0.850; TT-WW: *n* = 12, *Z* = 19.0, *P* = 0.151) and pupae to adult stages (Wilcoxon signed-rank tests: TW-WW: *n* = 12, *Z* =  −10.0, *P* = 0.457; TT-WW: *n* = 12, *Z* = 19.0, *P* = 0.151) developmental periods (Fig. 5B). However during development from L_1_ larvae to pupae the relative fitness of both the hemizygous (*Z* =  −27.0, *P* = 0.034) and homozygous EVida3 transgenics (*Z* =  −30.0, *P* = 0.016) were significantly reduced (Fig. 5B).

No significant difference in genotypic frequencies was found between the two pupal samples, indicating comparable developmental rates (Logistic regression: *n* = 722, replicate: df = 4, *χ* ^2^ = 5.8, *P* = 0.215; sample: df = 2, *χ* ^2^ = 2.48, *P* = 0.289).

## Discussion

We assessed the fitness of two *Anopheles gambiae s.s*. transgenic lines recently developed using a two-phase targeted genetic transformation system. The experimental design enabled us to detect initial differences in mating and reproductive success, assortative mating and hybrid vigor - all factors important in a future field-release scenario. In addition, the potential fitness costs of the unloaded Phase-1 and loaded Phase-2 genetic constructs, independent of the strains’ original genetic backgrounds, were estimated at the 1^st^ instar larvae, pupae and adult stages over the next 10 generations.

When we considered the performance of the unloaded, Phase-1 transgenic cassette (Mopti vs EE comparisons) over 10 generations, we found that it was stably integrated into our mixed population and achieved HWE in all replicates. Whilst we found no evidence for the potential confounding effects of differential fitness - in terms of male mating success, adult survival and female fertility - between Mopti and EE prior to recombination (F_0_–F_1_), and of hybrid vigor in the F_1_, we did observe a deficiency in hemizygotes in the F_1_ indicating some level of assortative mating. However this phenomenon had no effect on the outcome of the experiment, as in subsequent generations the frequency of hemizygotes became consistent with that predicted by HWE. This occurred despite evidence of decreased allelic fitness during the larval developmental stages. Genotypic fitness followed the same pattern albeit not significantly so - arguably because of the lower statistical power of the signed-rank test used. However we did observe a significant difference in genotypic fitness between hemi and homozygous transgenic individuals at the larval development stage. This genotypic fitness difference and decreased relative allelic fitness of the EE allele is unlikely to be due to expression of the ECFP phenotypic marker that is expressed co-dominantly and throughout all life stages - although an overdominance effect cannot be completely ruled out [20]. Thus, the most likely explanation is that this effect is a result of a recessive, weakly deleterious allele linked to the transgene insertion. Nevertheless this fitness cost, whilst observed consistently in all replicates, did not affect the eventual outcome of the experiment over time, as the effects were ameliorated by a higher (but not statistically significantly) fitness relative to wild-type measured between the adult to L1 larvae stages. Previous studies, albeit only considering adults at each generation, have also found similar, recessive fitness effects in otherwise stable transgenic strains. For example, despite reporting a transgenic strain of *An. stephensi* expressing the SM1 peptide being stable in mixed transgenic and non-transgenic cage invasion experiments [8], a later study investigating transgenics from the same strain, detected a homozygous fitness load [15].

In Mopti vs EVida3 comparisons we investigated the performance of the Phase-2 AMP-loaded transgene cassette (EVida3) and found that within 10 generations the transgene could not be detected either visually or through PCR analysis in any of the 3 replicates. This was despite observing that pre-recombination fitness parameters -i.e. the combination of F_0_ male and female mating success, adult survival and female fertility - were significantly higher than in the Mopti wild type. Further experiments should clarify which of these parameters is responsible for the higher initial reproductive success of the EVida3 strain.

Although we could not detect significant evidence of assortative mating in EVida3 vs Mopti comparisons (no hemizygote deficiency in the F_1_), we did observe a significant and immediate decrease in absolute fitness in both F_1_ homo- and hemizygotes. Due to the sharp drop in fitness of both these groups it was impossible to determine the effects (if any) of heterozygosity. Despite the rapid reduction in the frequency of the EVida3 transgenic cassette between the ensuing 10 generations, when we considered relative genotypic fitness within each generation we found that significant fitness costs were confined to the larval development sample. In contrast to Mopti vs EE comparisons, however, significantly reduced fitness was observed in both homozygous and hemizygous individuals. The fact that a fitness cost was observed in hemizygous EVida3 contrasts with the results found in a long-term stability studies of transgenic *A. stephensi* expressing SM1 under the control of the *Anopheles gambiae vitellogenin* promoter [16]. In those experiments, whilst hemizygotes persisted at high frequency (∼0.4) in the cage invasion populations, homozygous transgenic individuals were found at very low frequency (<0.1) suggesting a recessive fitness load [16].

Clearly the fitness costs imposed by the EVida3 construct at the larval stage, cannot be wholly explained by the fitness costs observed in the EE vs Mopti comparison - although these were likely to contribute to the lower fitness of homozygous larvae. Background expression of the *carboxypeptidase* promoter has been observed in adults of the EVida3 line outside of its post-bloodmeal expression profile [3]. Therefore, one possible explanation for this fitness load is that a low level of background expression of Vida3 leads to fitness costs that are detected only during the comparatively long growth interval between L1 larvae to pupae. Additionally, as with our EE vs Mopti comparisons, we cannot rule out dose-dependent toxicity of phenotypic markers. Increased apoptosis in cell lines carrying GFP and EGFP plasmids has been observed [20], and subsequent studies have indicated that prolonged excitation of fluorophores can increase the incidence of active oxygen species in neurones *in vitro* [21] and interrupt post-translational polyubiquitination in mice *in vivo* [22]. Finally it is possible that there is an independent deleterious effect caused by transgenic insert size (EE ∼4 kb, EVida3 ∼11 kb). There is some evidence from studies in *Drosophila* that fitness was reduced in individuals carrying larger (non-coding) transgenic inserts [23] relative to those carrying a smaller non-coding insert. Furthermore, transformation efficiency is reported to be inversely proportional to insert size in both *Drosophila* [24] and *An. gambiae* [3], which may indicate that larger transgenic constructs induce dominant deleterious effects through their size alone.

Whilst it is disappointing that EVida3 is uncompetitive and thus unlikely to be a strong candidate for a future transgenic release despite its demonstrated refractoriness to some *Plasmodium* infections [3], these results constitute an important proof of concept of the power of the site-specific two-stage transformation process. Furthermore, in the EE line, we have identified a fit competitive base on which to build, test and evaluate future Phase-2 transgenic lines and thus a powerful model system for evaluating the potential genetic load of candidate transgenic constructs carrying effector genes targeting the malaria parasite or other mosquito traits impacting malaria transmission.

## Materials and Methods

### Mosquito Strains and Insectary Conditions

The EE and EVida3 transgenic strains of *An. gambiae* [3] were used to assess the different sources of fitness costs potentially affecting transgenic lines. The Phase-1 EE strain carries a transgene cassette consisting of the phenotypic marker ECFP under the control of the 3xP3 promoter driving its expression in the eyes and other nerve tissues, and the *phiC31* integrase recognition sequence *attP* [18]. The Phase-2 EVida3 strain derived from the EE strain in a second transformation step carries a cassette consisting of 3xP3:ECFP, an additional marker 3xP3:*Ds*Red2 and the synthetic AMP Vida3 sequence with the *An. gambiae carboxypeptidase* promoter, signal peptide and UTRs [3]. The docking site is situated on chromosome 3R (position 15801959 - band 31B) and is therefore located away from any of the inversion polymorphisms commonly found in *An. gambiae* s.s. [25], [26]. The two transgenic lines were derived from the wild-type strain KIL originally colonized from Tanzania in the 1970’s. Both transgenic strains are of the M molecular form [27]. The wild-type strain used in this experiment is a Mopti, M-form population originally colonized from the village of N’Gabakoro Droit, Mali in 2003. Since it has been in our laboratory, the Mopti strain has been refreshed yearly by outcrossing to the F_1_ of field caught individuals from the same site. Both transgenic stocks were maintained as true-breeding homozygotes and, along with wild-type strains, were kept in dedicated insectaries at 27±2°C, 70±5% relative humidity, with a 12 h light/dark cycle. Larvae were grown at a density of 200 larvae/l and fed an optimized regimen of ground fish food (Tetramin). Upon pupation, pupae were transferred to a standard rearing cage made of a 5 l white polypropylene bucket (∼20.5 cm height × 20 cm diameter) with a sleeved side opening for introducing and removing mosquitoes and accessories, and the top covered with mosquito netting. Adults were typically maintained at densities of 600–800 adults per enclosure and provided with water and a 5% glucose solution *ad libitum*.

### Cage Invasion Experiments

Cage invasion experiments were initiated by mixing 100 male and 100 female homozygous wild-type mosquitoes (WW) with 100 male and 100 female homozygous transgenic mosquitoes (TT). All individuals were 3–5-days old and unmated prior to mixing. After allowing 2 dark cycles for mating, mosquitoes were bloodfed to produce eggs and, after a further 2 d, provided with a ∼10 cm diameter pot lined with wet filter paper (grade 1, Whatman) for oviposition. Eggs were hatched in 1 l of ddH_2_O and randomly selected L_1_ larvae separated into 6 growth trays per experimental replicate at a density of 200 larvae/l resulting in population sizes ∼900–1100 individuals at each generation. Larvae were maintained in the same conditions as the stock populations (see above). Once pupated, individuals were transferred to a standard 5 l adult enclosure to emerge. Adult were maintained in the same conditions as the stock populations (see above) and left to mature and mate. Four days after adding the last pupae to the cages, adult females were bloodfed to produce the next generation. Mixed populations were maintained in this way for 10 generations.

### Sampling

The frequency of the transgene was determined at three key life stages: first instar L_1_ larvae, pupae (2 samples taken on the 2^nd^ and 5^th^ day of pupation), and 2-day post-emergence adults. At each life stage, 48 individuals were selected at random from each population and genomic DNA was extracted using a modified DNAzol gDNA extraction protocol (Invitrogen). The two samples of pupae taken at a 3-day interval were used for detecting potential differences in developmental rate between genotypes. Transgenic status was then determined by carrying out a PCR on the extracted DNA using primers designed to produce characteristic gel bands for homozygous transgenic (TT), homozygous wild type (WW) or a hemizygous hybrid (TW) (Table 1). Hence the precise genotypic and allelic frequencies could be calculated for each life stage.

**Table 1:** Primer sequences and amplicon size (bp) for genotyping in cage-invasion experiments.

Primers	Sequence (5′-3′)	Genotype	Size (bp)
Uni_Fwd	CCATCCCCAAAAAAATGAACTGAAA	–	–
Mopti_Rev	TCCCTCTTATAAGTAAGGGTTGC	WW	172
E_Rev	GCAGACTATCTTTCTAGGGTTAAACTG	TT	166

A universal forward primer was combined with reverse primers specific to the Mopti or transgenic EE and EVida3 lines to generate diagnostic bands in two independently-run PCR reactions (see methods for details).

### Data Analyses

#### Mating, reproductive success and hybrid vigor in the initial generation (F0–F1)

Assortative mating/hybrid deficiencies in comparisons of Mopti vs EE and Mopti vs EVida3 were tested by comparing the observed frequency of hybrids and homozygous genotypes in the L_1_ larvae sample of the F_1_ progeny to the 50∶50 ratio predicted given the equal numbers of WW and TT males and females used to initiate each experiment using Chi-square Goodness of Fit tests. Similarly, the reproductive success of transgenic and non-transgenic lines was assessed prior to any recombination events by comparing the frequencies of transgenic and wild-type alleles in the F_1_ progeny (L_1_ Larvae in both experiments) using Chi-square Goodness of Fit tests. Finally, the effects of heterozygosity or hybrid vigor on survival from larval to pupal and from pupal to adult stages were tested by comparing the change in allelic frequencies of hemizygotes and homozygotes TT and WW from the F_1_ L_1_ larvae to the F_1_ adults stages using Chi-square of Association tests. Post-hoc pairwise frequency comparisons were conducted using the Marascuilo procedure.

#### Transgenic vs wild-type frequencies comparisons (F1–10)

Potential fitness costs associated with the transgene in generations F_1–10_ were assessed by monitoring allele and genotype frequencies over time. Deviations from the predicted HWE ratios from one generation to the next were used for detecting selection against certain genotypes [28]. Based on the starting conditions (100 males and 100 female homozygous wild-type (WW) and 100 males and 100 female homozygous TT of either Phase-1 EE or Phase-2 EVida3) and assuming random mating and no fitness costs on the transgenic strains and transgenic allele, the expected Mendelian genotypic frequencies are 0.25 for homozygote WW and TT and 0.50 for hemizygous TW individuals. Deviations from those ratios and those predicted by HWE were tested using Chi-square Goodness of Fit tests.

#### EE vs EVida frequencies comparisons (F1–10)

The overall and generation-by-generation frequencies of EE and EVida3 transgenic alleles competing against the ‘wild-type’ Mopti allele in EE vs Mopti and EVida3 vs Mopti comparisons were formally compared across both type of comparisons and all replicates using multivariate logistic regression.

#### Life stage-specific fitness costs (F2–F5)

In order to enable more precise statistical comparisons of the performance of the EE, EVida3 and wild-type alleles across the EE and EVida3 vs Mopti comparisons, we calculated their genotypic and allelic fitness [28], [29] relative to that of the Mopti wild-type allele. Fitness was not only calculated between generations but also broken down into fitness components between sampling within generations in order to highlight selection acting against alleles and genotypes at different life stages [29].

First, the Absolute genotypic fitness (*W*_(abs)_) was estimated as the change in frequency (f) of a given genotype over time, either between generations or between samples:
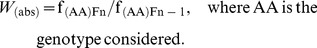
(1)


Similarly, absolute allelic fitness was calculated as:
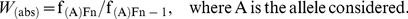
(2)


Allelic and genotypic fitness relative to the wild-type strain *W*_(rel)_ were calculated and plotted in graphs as the absolute fitness *W*_(abs)_ normalized by dividing it by the absolute fitness of the wild-type strain *W*_(abs WW)_:
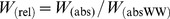
(3)


The relative genotypic and allelic fitness *W*_(rel)_ between generations and between samples within each generation was calculated from the differences in genotypic frequencies observed in generations F_2_–F_5_ following (Eq. 1, 2 and 3), but using between-stage changes rather than between generations ones. After checking the data for deviations from normality, Wilcoxon signed-rank tests were used to compare values of relative allelic fitness *W*_(rel)_ of the EE and EVida transgenic alleles against the base line of the Mopti wild-type allele (relative fitness = 1). Wilcoxon signed-rank tests were also used to compare the fitness of hemi and homozygous transgenic individuals against the base-line wild-type homozygous ones. Comparisons between transgenic allele type (EE or EVida3) or hemi and homozygous transgenic genotypes were performed using Mann-Whitney tests.

All statistical analysis and graphing were carried out using JMP (SAS Institute inc.). Significant differences between replicates were checked in all analyses and reported whenever appropriate.

## Supporting Information

Table S1 **Genotypic frequency over time, EE vs Mopti comparison.** The frequency of individuals homozygous and hemizygous for the Phase-1 EE construct and homozygous wild-type individuals over 10 generations in three replicates.
(XLS)

Table S2 **Genotypic frequency over time, EVida3 vs Mopti comparison.** The frequency of individuals homozygous and hemizygous for the Phase-2 EVida3 construct and homozygous wild-type individuals over 10 generations in three replicates.
(XLS)
